# A Large-Scale Comparative Metagenomic Study Reveals the Functional Interactions in Six Bloom-Forming *Microcystis*-Epibiont Communities

**DOI:** 10.3389/fmicb.2018.00746

**Published:** 2018-04-20

**Authors:** Qi Li, Feibi Lin, Chen Yang, Juanping Wang, Yan Lin, Mengyuan Shen, Min S. Park, Tao Li, Jindong Zhao

**Affiliations:** ^1^State Key Laboratory of Freshwater Ecology and Biotechnology, Institute of Hydrobiology, Chinese Academy of Science, Wuhan, China; ^2^University of Chinese Academy of Sciences, Beijing, China; ^3^Oil Crops Research Institute of the Chinese Academy of Agricultural Sciences, Wuhan, China; ^4^Center for Microalgal Biotechnology and Biofuels, Institute of Hydrobiology, Chinese Academy of Science, Wuhan, China; ^5^State Key Laboratory of Protein and Plant Genetic Engineering, College of Life Sciences, Peking University, Beijing, China

**Keywords:** cyanobacterial blooms, metagenomics, *Microcystis*, epibionts, microbe-microbe interactions, environmental microbiomes

## Abstract

Cyanobacterial blooms are worldwide issues of societal concern and scientific interest. Lake Taihu and Lake Dianchi, two of the largest lakes in China, have been suffering from annual *Microcystis*-based blooms over the past two decades. These two eutrophic lakes differ in both nutrient load and environmental parameters, where *Microcystis* microbiota consisting of different *Microcystis* morphospecies and associated bacteria (epibionts) have dominated. We conducted a comprehensive metagenomic study that analyzed species diversity, community structure, functional components, metabolic pathways and networks to investigate functional interactions among the members of six *Microcystis*-epibiont communities in these two lakes. Our integrated metagenomic pipeline consisted of efficient assembly, binning, annotation, and quality assurance methods that ensured high-quality genome reconstruction. This study provides a total of 68 reconstructed genomes including six complete *Microcystis* genomes and 28 high quality bacterial genomes of epibionts belonging to 14 distinct taxa. This metagenomic dataset constitutes the largest reference genome catalog available for genome-centric studies of the *Microcystis* microbiome. Epibiont community composition appears to be dynamic rather than fixed, and the functional profiles of communities were related to the environment of origin. This study demonstrates mutualistic interactions between *Microcystis* and epibionts at genetic and metabolic levels. Metabolic pathway reconstruction provided evidence for functional complementation in nitrogen and sulfur cycles, fatty acid catabolism, vitamin synthesis, and aromatic compound degradation among community members. Thus, bacterial social interactions within *Microcystis*-epibiont communities not only shape species composition, but also stabilize the communities functional profiles. These interactions appear to play an important role in environmental adaptation of *Microcystis* colonies.

## 1. Introduction

Cyanobacteria constitute a significant part of the primary producers in most water ecosystems and play an important role in global carbon and nitrogen cycles (Garcia-Pichel et al., 2003; Elbert et al., 2012). Harmful cyanobacterial blooms are a worldwide problem of societal concern and scientific interest (Falconer, 2001; Hilborn et al., 2014; Otten and Paerl, 2015) and annually recur in many inland, estuarine, and coastal eutrophic waters (Morel and Price, 2003; Paerl and Otten, 2013).

Among bloom-forming cyanobacteria, *Microcystis* is the most frequently reported genus responsible for freshwater cyanobacterial blooms (Visser et al., 2005). *Microcystis* colonies harbor numerous epibionts that are sheltered from grazing by being embedded in the *Microcystis* mucilage (Brunberg, 1999). Since *Microcystis* is a dominant species that causes harmful blooms, the majority of early studies of *Microcystis*-based blooms were focused on the phylogenetic classification of *Microcystis* species and the environmental factors that influence *Microcystis* growth (Oliver and Ganf, 2000; Davis et al., 2009). Recently, researchers have started to pay attention to the microbial community structure of *Microcystis* colonies.

To investigate community structure, species diversity, and *Microcystis* and epibiont identities, various molecular techniques were previously used, including enzyme assays, 16S rRNA sequence-based phylogeny, and denaturing gradient gel electrophoresis (DGGE) (Kapustina, 2006; Limei et al., 2009; Shi et al., 2010). These studies identified Alphaproteobacteria, Betaproteobacteria, and Gammaproteobacteria as the predominant bacteria that are associated with various *Microcystis* species (Shi et al., 2010; Parveen et al., 2013). Significantly, some previous studies revealed close interactions between *Microcystis* and epibionts. For example, phosphorus exchange between *Microcystis aeruginosa* and associated *Pseudomonas* spp. was observed in the phycosphere (Jiang et al., 2007). Additionally, the cyanopeptides in *Microcystis* exudates provide dissolved organic carbon and nutrients that are needed for growth of other bacteria (Briand et al., 2016). These reports on bacterial social activities have generated great interest in studying community-based interactions of the *Microcystis* microbiome.

The molecular techniques used in the studies described above produced a large body of useful information on community structure and diversity, but limited detailed information on interactions among *Microcystis* colony members. These techniques also suffer from insufficient resolution in identification of the member bacteria at species level. More importantly, these techniques fail to produce comprehensive datasets that are essential for identifying functional components or building complete metabolic networks. Without such information, it becomes difficult to visualize functionally meaningful interactions between *Microcystis* and epibionts. Metagenomics provides a large amount of genetic information that can help researchers decipher the interactions within a microbial community. It allows us to gain insights into epibionts strategies for successful environmental adaptation without the need to culturing (Debroas et al., 2009; Li et al., 2011; Steffen et al., 2012). Furthermore, modern microbiology has been transformed by the metagenomic-based study of microbiomes because of the advantages of high-throughput technologies (Marchesi and Ravel, 2015). The conceptual framework of microbiomes can provide a novel insight into microbial communities, which are more than the sum of the individual members. The functional complementation, dynamic shuffling of genetic information, and close communication among community members are hallmarks of microbiomes, such as gut microbiomes (Gill et al., 2006; Human Microbiome Project Consortium, 2012).

In this study, we used *Microcystis* colonies isolated from Lake Taihu and Lake Dianchi. Both lakes suffer from major cyanobacterial blooms every year. Lake Taihu is the third largest freshwater lake in China and is located in the mid-lower Yangtze Plain, which has been experiencing rapid economic development and population growth for the last 30 years (Qin et al., 2007). In Lake Taihu, harmful cyanobacterial blooms are rampant due to severe discharges from agricultural, livestock, municipal, and industrial wastewater to tributaries and rivers, and also to the lack of sufficient wastewater treatment facilities. Lake Dianchi is the sixth largest and one of the most heavily eutrophic lakes in China (Wu et al., 2010). Because Lake Dianchi has only one outlet with several rivers flowing into the lake, overloaded nutrients persist in the lake (Zhu et al., 2013). Regional surveys and other studies have shown that three *Microcystis* morphospecies (*M. aeruginosa, M. wesenbergii*, and *M. flos-aquae*) were the dominant blooming species in both Lake Taihu and Lake Dianchi (Yu et al., 2007; Chen et al., 2009).

We used comparative metagenomics to investigate the species diversity, community structure, and functional interactions between *Microcystis* morphospecies and epibionts isolated from Lake Taihu and Lake Dianchi. We addressed the following key questions: (1) Who are the major components in *Microcystis*-epibiont communities?; (2) What kind of functional interactions exist in *Microcystis*-epibiont microbiomes?; (3) Is there functional complementation in *Microcystis*-epibiont microbiomes?; (4) Are the community structures varying with the morphospecies or the origin?; (5) Based on the answers to the previous questions, are *Microcystis*-epibiont microbiome traits related to the environment where they originated? By elucidating these issues, we will gain an in-depth understanding of the interactions among the members of *Microcystis*-epibiont communities.

## 2. Materials and methods

### 2.1. Sample collection

**Figure 1:**
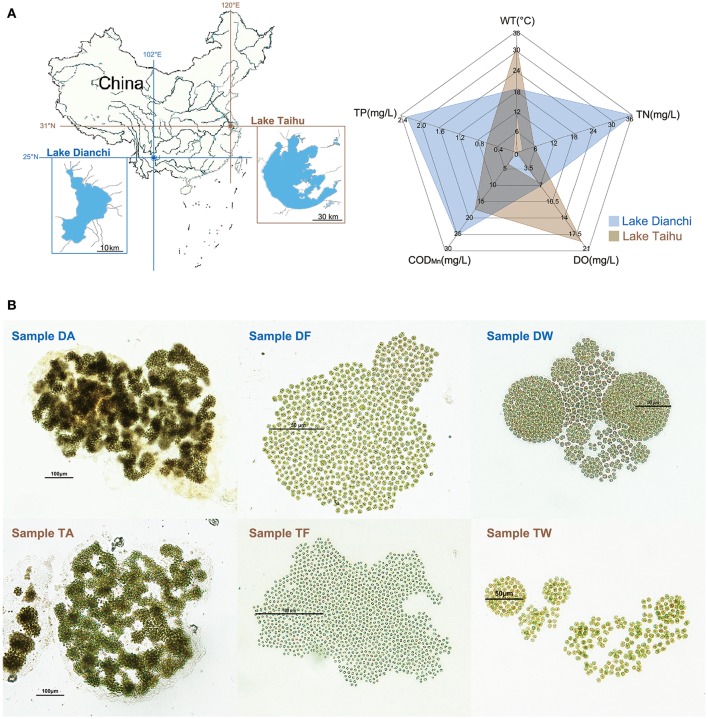
Sampling information. **(A)** Sketch map showing basic information of the study area. Left: locations of Lake Taihu and Lake Dianchi in China. Right: environmental characteristics of Lake Taihu and Lake Dianchi, including water temperature (WT), dissolved oxygen (DO), total nitrogen (TN), total phosphorus (TP), and chemical oxygen demand (COD_*Mn*_). The environmental data were based on previously reported data of Lake Dianchi and Lake Taihu in 2008 to 2009 (Huang et al., 2010; Niu et al., 2011; Cao et al., 2012), which was the same period during which sampling began. **(B)** Micrographs of six *Microcystis*-epibiont communities. Samples DA, DF, and DW were collected from Lake Dianchi, whereas samples TA, TF, and TW were collected from Lake Taihu; DA and TA, *M. aeruginosa*; DF and TF, *M. flos-aquae*; DW and TW, *M. wesenbergii*.

*Microcystis* colonies were sampled from hypereutrophic areas of Lake Dianchi (Yunnan Province, China) and Lake Taihu (Jiangsu Province, China) (Figure 1A and Supplementary Table S1). Single colonies with typical morphological characteristics of *M. aeruginosa, M. flos-aquae*, or *M. wesenbergii* were selected as described by Werner et al. (2015) and Komárek and Komárková (2002). The free-living bacteria were removed from each colony following the method described by Shi et al. (2010). Briefly, each colony was washed 10 times on autoclaved nylon screen (pore size, 20μm) with autoclaved phosphate buffered saline. The associated bacteria remained within the mucilage as members of *Microcystis*-epibiont communities. We cultivated six *Microcystis*-epibiont communities as unicyanobacterial xenic cultures for a few months to 3 years from original isolation. The cultures were maintained by serial subculture, aseptically, every 3 weeks on liquid BG-11 medium. All cultures were incubated at 25°C with a photon irradiance of 25μmol · m^−2^ · *s*^−1^ with a 12/12-h light/dark cycle.

### 2.2. DNA extraction and sequencing

For DNA isolation, colonies from each community were harvested in the exponential growth phase by centrifugation. Community genomic DNA extraction was conducted using a phenol-chloroform protocol modified from Xie et al. (2016). A paired-end library with insert sizes of 300 bp and a mate-paired library with insert sizes of 3 Kb were constructed for each community genomic DNA sample according to instructions from Illumina. The libraries were sequenced on an Illumina Genome Analyzer IIx. DNA preparations were also sent for sequencing on a Pacific Biosciences (PacBio) RS sequencing platform to close or improve captured gaps.

### 2.3. Metagenome assembly and binning

Quality trimming of raw sequence reads was performed by Trimmomatic v.0.36 (Bolger et al., 2014) with leading or trailing bases quality score of 10, four-base window threshold of 20, and minimum length of 20 bp. *De novo* assembly of each sample sequence was conducted with SPAdes v.3.8.2 (Bankevich et al., 2012) with a minimum contig length of 200 bp and minimum average coverage of 3. Scaffold average coverage was calculated by the genomeCoverageBed command in BEDTools (Quinlan and Hall, 2010).

To cluster scaffolds into individual genome bins, we used a visualization-guided binning method based on both GC content and read coverage of each scaffold (Xie et al., 2016), with some modifications. Specifically, if a non-clustered fragment contained a gene from the set of 107 essential single-copy genes that was conserved in 95% of all sequenced bacterial genomes (Dupont et al., 2012), the original method would draw back the fragment from the unassigned sequences; instead, we set a threshold for each sample guided by the graph of scatter points to select the non-clustered fragments containing essential single-copy genes, rather than selecting all of the fragments. In addition, the distribution of essential single-copy genes was considered as a reflection of genome completeness and contamination. Moreover, we used CheckM v1.0.7 (Parks et al., 2015) to validate the accuracy of completeness and contamination estimates for each genome bin.

### 2.4. Taxonomic classification

Open reading frames (ORFs) of each genome bin were predicted by Prodigal v2.6.3 in metagenomic mode. We implemented taxonomic assignments of the genome bins using TAXAassign v.0.4 (https://github.com/umerijaz/taxaassign), with some modified codes to improve efficiency and accuracy. Using the improved script, we used deduced amino acid sequence information through DIAMOND BLASTP (Buchfink et al., 2015) searches, instead of nucleotide sequences through BLASTN searches, to produce a protein sequence alignment against the NCBI non-redundant (nr) protein database with parameters *E*-value ≤ 1*e*−5, sequence identity > 60%, query coverage > 50%, and number of matches reported ≤ 100.

The protein-encoding genes within the same genome bin were not always assigned to the same taxon, which made it difficult to assign each genome bin to a specific taxon. To address this problem, we selected the taxon that had the most assigned sequences within the same bin accorded to as the main taxon at the family or genus level. The specific taxonomic classification rules are shown in Supplementary Figure S1. For each genome bin, all protein sequences were initially assigned at the genus level, and the taxon with highest abundance was considered as the main taxon of this genome bin. The abundance of the main taxon was defined as the CONSISTENCY of the taxonomic assignment. We only considered a taxon with consistency of >65% as the taxonomic identification of the bin.

### 2.5. Genome bin evaluation

Because genomes reconstructed from metagenomic data usually vary substantially in quality, we proposed a set of quality criteria with quantitative thresholds to evaluate genome quality for subsequent analyses (Table 1). Contig number, genome completeness, genome contamination, and taxonomic assignment consistency (as mentioned above) were considered as part of the quality criteria, which was revised from previous standards (Chain et al., 2009; Parks et al., 2015; Güllert et al., 2016). We used the quality criteria to evaluate the genome bins with a rating, and selected high-quality bins for genome reconstruction.

**Table 1:** Quality criteria for evaluation of genome bins.

**Bin category**	**Genome classification**	**Rating**	**Quality criteria**
High-quality bins	Nearly complete genomes	*****	95% completeness, 5% contamination, 90% consistency, and 10 contigs
			Or 90% completeness, 5% contamination, 80% consistency and 3 contigs
	Good genome drafts	****	90% completeness, 5% contamination, and 80% consistency
Medium-quality bins	Moderate genome drafts	***	70% completeness, 10% contamination, and 65% consistency
Low-quality bins	Partial genome or pangenome drafts	**	50% completeness, 15% contamination, and 65% consistency
Disqualified bins	Mixed or fractional genome sequences	*	Not meeting any sets of criteria above

### 2.6. Phylogenetic analysis

To infer phylogenetic relationships among bacteria, a whole-genome-based and alignment-free Composition Vector Tree (CVTree) method (Xu and Hao, 2009) was used to compare and cluster the genomes we extracted from our assemblies. Distances were computed using the composition vector representation of amino acid sequences. The distance matrix was used to construct phylogenetic trees by the neighbor-joining method in PHYLIP v3.696. Moreover, to validate the taxonomic assignment of high-quality bins, phylogenetic analyses were undertaken within the genera to which the genome bins were assigned based on both whole-genome sequences and 16S rRNA genes.

### 2.7. Functional annotation and metabolic pathway reconstruction

Functional characterization and annotation of protein-encoding genes were performed by MOCAT2 (Kultima et al., 2016). The protein-encoding genes of each sample were separately submitted to the automatic annotation server GhostKOALA (last updated March 4, 2016) (Kanehisa et al., 2016) to analyze individual gene function and Kyoto Encyclopedia of Genes and Genomes (KEGG) pathways (Kanehisa and Goto, 2000). Furthermore, orthologous protein sets from different samples were identified and gathered as clusters of orthologous groups (COGs) with differential abundance by the application of the basic local alignment search tool (BLAST) (Altschul et al., 1990) and MEGAN v5.11.3 (Huson et al., 2016).

### 2.8. Data and material availability

Raw metagenome sequence reads in this study were deposited in the NCBI Sequence Read Archive under accession numbers SAMN06925823, SAMN06925961, SAMN06925965, SAMN06925966, SAMN06925967, and SAMN06925977. Source codes, example softwares, and genomic data are available at https://github.com/qi-lee/Meta-Microbiome/.

## 3. Results

### 3.1. Sampling, sequencing, and assembly

We isolated and subcultured six samples of *Microcystis*-epibiont communities from Lake Dianchi and Lake Taihu. Based on previously reported data (Huang et al., 2010; Niu et al., 2011; Cao et al., 2012), the environmental characteristics of Lake Dianchi and Lake Taihu are displayed in Figure 1A. The total nitrogen (TN) and total phosphorus (TP) in Lake Dianchi were higher than those in Lake Taihu, whereas water temperature (WT) and dissolved oxygen (DO) varied greatly in Lake Taihu. Three *Microcystis* morphospecies were distinguished based on morphological characteristics, such as margin of colonies, patterns of arrangement, size of cells, and form of mucilage (Figure 1B). We labeled the samples (DA, DF, DW, TA, TM, and TW) by their original locations (D: Lake Dianchi, T: Lake Taihu) and dominant morphospecies (A: *M. aeruginosa*, F: *M. flos-aquae*, W: *M. wesenbergii*) (Supplementary Table S1).

Whole-genome shotgun sequencing of the six samples generated approximately 640 million raw reads (>56.5 Gb of sequences). After trimming low-quality reads, 75% of raw reads were retained as clean data (493,065,004 total reads). The clean data were assembled using the SPAdes Genome Assembler and generated 111,324 scaffolds for which N50 ranged from 11.1 to 204.2 Kb. The total length of the six assemblies ranged from 39.5 to 70.7 Mb. More details about sequencing and assembly for each of the samples are presented in Supplementary Table S2.

### 3.2. Binning process and taxonomic assignment

A visualization-guided binning protocol was used, and each bin was plotted in different colors (Supplementary Figure S2). Since genomes reconstructed from metagenomic data usually vary substantially in quality, we proposed a set of quality criteria with quantitative thresholds to evaluate the genome quality for subsequent analyses (Table 1). Contig number, genome completeness, genome contamination, and taxonomic assignment consistency (see section 2) were considered in the quality criteria, as a revision of previous standards (Chain et al., 2009; Parks et al., 2015; Güllert et al., 2016). Sixty-eight genome bins were extracted from the assemblies (14 bins from sample DA, 17 bins from sample DF, nine from sample DW, nine from sample TA, nine from sample TF, and 10 from sample TW).

The completeness of each genome bin was estimated by manual calculation based on the presence of the 107 essential single-copy genes mentioned above and produced completeness values that varied between 33.64 and 99.07% (mean 86.06%). Verification by CheckM produced similar outputs that ranged from 39.75 to 100% (mean 87.47%). We successfully recovered 43 genome bins with completeness >90%, which indicated good potential genomes. The basic statistics of each genome bin are shown in Table 2, and more details are provided in Supplementary Table S3.

**Table 2:** Basic information on the 68 genome bins extracted from metagenomic data.

**Genome Bin ID**	**Genome Bin Size (Mb)**	**Relative abundance (%)**	**Taxonomic assignment**	**Quality rating**
DA01	4.69	0.53	*Bosea*	*
DA02	3.34	0.50	*Rhizobium/Agrobacterium*	*
DA03	2.59	0.54	*Porphyrobacter*	**
DA04	2.73	0.58	*Methylophilus*	****
DA05	3.70	0.97	*Hyphomonadaceae*	****
DA06	6.66	2.00	*Rhodobacter*	*
DA07	3.48	1.73	*Rhizobium/Agrobacterium*	***
DA08	4.66	2.15	*Gemmatimonas*	***
DA09	3.82	2.72	*Hyphomonas*	******
DA10	3.37	4.32	*Porphyrobacter*	****
DA11	2.79	5.99	*Brevundimonas*	***
DA12	5.14	12.28	*Pseudomonas*	****
DA13	3.02	10.18	Unclassified	*****
DA14	5.14	55.50	*Microcystis*	****
DF01	2.11	0.14	Unclassified	**
DF02	2.42	0.42	*Pseudoxanthomonas*	**
DF03	1.59	0.28	*Xanthomonadaceae & Burkholderiaceae*	*
DF04	3.42	1.05	*Bacterium YEK0313*	***
DF05	3.57	1.11	*Hyphomonadaceae*	****
DF06	2.42	1.03	*Brevundimonas*	***
DF07	3.38	1.52	*Hydrogenophaga*	***
DF08	5.84	2.83	*Mesorhizobium*	***
DF09	4.71	2.43	*Acidovorax*	*
DF10	3.81	2.40	*Rhodobacter*	***
DF11	3.03	1.50	*Sphingomonadaceae*	*****
DF12	4.76	3.26	*Xanthomonadaceae*	****
DF13	4.63	8.40	*Rhizobium/Agrobacterium*	****
DF14	3.37	6.14	*Silanimonas*	*****
DF15	2.64	12.06	*Brevundimonas*	*****
DF16	3.27	19.01	*Limnobacter*	*****
DF17	4.93	36.39	*Microcystis*	****
DW01	2.79	1.41	*Elstera*	*
DW02	5.59	3.41	*Acidovorax*	*
DW03	3.49	2.98	*Hyphomonadaceae*	***
DW04	4.30	6.89	*Rhodobacter*	**
DW05	3.19	4.45	*Sphingomonadaceae*	*****
DW06	2.74	8.71	*Silanimonas*	***
DW07	2.61	7.03	Unclassified	*****
DW08	3.00	18.80	*Flavobacterium*	****
DW09	4.55	46.32	*Microcystis*	****
TA01	2.13	0.48	*Limnobacter*	**
TA02	7.20	1.66	*Variovorax*	*
TA03	3.68	1.31	*Bosea*	**
TA04	4.05	6.97	*Pseudoxanthomonas*	****
TA05	3.31	9.80	*Limnobacter*	****
TA06	4.38	18.02	*Rhizobium/Agrobacterium*	****
TA07	2.57	6.58	*Xanthomonadaceae*	**
TA08	2.71	13.34	*Brevundimonas*	**
TA09	5.01	41.84	*Microcystis*	****
TF01	1.18	0.26	*Limnobacter*	*
TF02	6.02	1.32	*Comamonadaceae & Phyllobacteriaceae*	*
TF03	3.45	1.15	*Pseudoxanthomonas*	****
TF04	5.27	2.02	*Rhizobium/Agrobacterium*	*
TF05	4.92	2.27	*Pseudomonas*	****
TF06	4.53	7.42	*Rhizobium/Agrobacterium*	****
TF07	3.05	11.14	*Sphingopyxis*	***
TF08	2.98	10.03	*Brevundimonas*	****
TF09	5.09	64.40	*Microcystis*	****
TW01	3.27	0.69	*Flavobacterium*	****
TW02	1.85	0.59	*Limnobacter*	**
TW03	1.00	0.26	*Hyphomonas*	*
TW04	6.05	1.72	*Pseudoxanthomonas*	*
TW05	3.88	3.49	*Bosea*	***
TW06	4.36	7.17	*Rhizobium/Agrobacterium*	****
TW07	2.94	5.05	*Limnobacter*	***
TW08	4.56	10.29	*Pseudomonas*	****
TW09	4.73	15.79	*Flavihumibacter*	*****
TW10	4.56	54.96	*Microcystis*	****

ORFs of each bin were predicted, and a total of 261,242 protein-encoding genes were extracted. In taxonomic classification based on protein-encoding genes, 35.61% of the sequences (93,028/261,242) were successfully assigned at the genus level, and 23.95% (62,575 sequences) were assigned at the species level. Additionally, 55 genome bins were unambiguously identified at the genus level with >65% consistency. Seven genome bins were identified at the family level, affiliating with the phyla Cyanobacteria (class Oscillatoriophycideae), Proteobacteria (classes Alphaproteobacteria, Betaproteobacteria, and Gammaproteobacteria), Gemmatimonadetes (class Gemmatimonadetes), and Bacteroidetes (classes Flavobacteriia and Chitinophagia), respectively (Supplementary Table S3). In addition, genome bin DF04 was identified at the species level as bacterium YEK0313 (Taxonomy ID: 1522316), which is reported as *Rasbobacterium massiliensis* sp. nov. of unknown lineage. The other five genome bins could not be classified to taxa in this way, ascribed to divergence of classification causing low consistency (for TF02, DF01, and DF03) or absence of protein-encoding genes that had matches in the NCBI nr database (for DA13 and DW07). Table 2 lists the taxonomic assignment of the 68 genome bins.

### 3.3. Genome reconstruction, evaluation, and annotation

All genome bins were measured by the quality criteria we proposed (Table 1) and are listed with rating markers in Table 2. We recognized 34 high-quality bins in this study (including nine nearly complete genomes and 25 good genome drafts). Among the nine nearly complete genomes, DA09, DF14, DF15, DF16, and TW09 were assigned to the genera *Hyphomonas, Silanimonas, Brevundimonas, Limnobacter* and *Flavihumibacter*, respectively, whereas DF11 and DW05 were both assigned to the family Sphingomonadaceae. The other two nearly complete genomes, the high-quality but unassigned bins DA13 and DW07, were exceptional cases; for both, genome completeness was high (>90%) and contamination was low (<1%), which indicates that they are good-quality genome bins. However, none of the protein-encoding genes within the bins were assigned to any taxon in the database (100% consistency), which led to no identification of either bin. We suggest that genome bins DA13 and DW07 represent genomic information of species without previous records. Another piece of evidence that supports our suggestion is the lack of closely related 16S rRNA gene sequences for DA13 or DW07 (only 87.91 and 87.01% identity, respectively, with the closest sequences in the NCBI 16S ribosomal RNA database). After confirming classification of the nearly complete genomes by clustering to species within assigned genera, both by whole-genome CVTree and 16S rRNA gene analyses, we submitted the genomes to GitHub. The genomic characteristics and accession details of the nine genomes are presented in Table 3.

**Table 3:** Basic statistics for the high-quality genomes obtained in this study.

**Strain name**	**Length**	**Total of contigs**	**N50**	**GC content**	**Number of genes**
*Hyphomonas* sp. DA09	3,818,430	5	899074	62.45	3,647
Unclassified bacterium DA13	3,023,577	1	3023577	60.26	2,875
*Sphingomonadaceae* sp. DF11	3,025,573	2	2471200	56.47	2,867
*Silanimonas* sp. DF14	3,369,458	4	1190267	69.32	3,001
*Brevundimonas* sp. DF15	2,639,421	2	2219995	69.42	2,635
*Limnobacter* sp. DF16	3,272,399	9	836353	52.50	3,066
*Sphingomonadaceae* sp. DW05	3,186,008	4	619893	56.56	3,010
Unclassified bacterium DW07	2,612,545	2	1739781	61.97	2,577
*Flavihumibacter* sp. TW09	4,727,931	1	4727931	41.23	4,092
*Microcystis aeruginosa* DA14	5,140,879	20	529925	42.74	4,831
*Microcystis flos-aquae* DF17	4,931,346	96	91151	42.89	4,725
*Microcystis wesenbergii* DW09	4,554,317	58	166848	43.26	4,270
*Microcystis aeruginosa* TA09	5,012,749	173	83551	42.69	4,853
*Microcystis flos-aquae* TF09	5,100,068	13	584877	42.41	4,959
*Microcystis wesenbergii* TW10	4,556,116	68	156945	43.28	4,291

It is worth mentioning that the six *Microcystis* genomes were also reconstructed as high-quality bins. Because of the high read coverage and distinctive GC content, it was easy to distinguish the *Microcystis* genome sequences from the associated bacterial genome sequences. Compared with previously published *Microcystis* genomes, these six genomes show a high level of intragenus similarity in terms of genome size, GC content, and sequences. We uploaded two *M. aeruginosa* assemblies, two *M. flos-aquae* assemblies, and two *M. wesenbergii* assemblies in GitHub. The characteristics of the six *Microcystis* genomes are presented in Table 3.

With effective annotation of the protein-encoding genes in the six samples, 229,822 genes (87.97%) had a protein domain annotated in the eggNOG database and 211,211 genes (80.85%) in Pfam, 141,226 genes (54.06%) had a match for metabolic pathways in the KEGG database, and 1,839 genes (0.7%) were annotated in the antibiotic resistance database. There were 31,420 genes (12.03%) that were previously unknown in the MOCAT2 integrated databases.

### 3.4. Overview of *Microcystis*-epibiont community species diversity and structure

As fundamental species of *Microcystis*-epibiont communities, *Microcystis* (order Chroococcales) was the most abundant group in each sample, with relative abundance ranging from 36.39 to 64.39% (Supplementary Figure S3). Aside from *Microcystis*, the six communities were composed of different epibionts. Each of the six *Microcystis*-epibiont communities contained bacteria affiliated with the classes Alphaproteobacteria, Betaproteobacteria, and Gammaproteobacteria, and some of the communities had other species related to the class Gemmatimonadetes and phylum Bacteroidetes.

**Figure 2:**
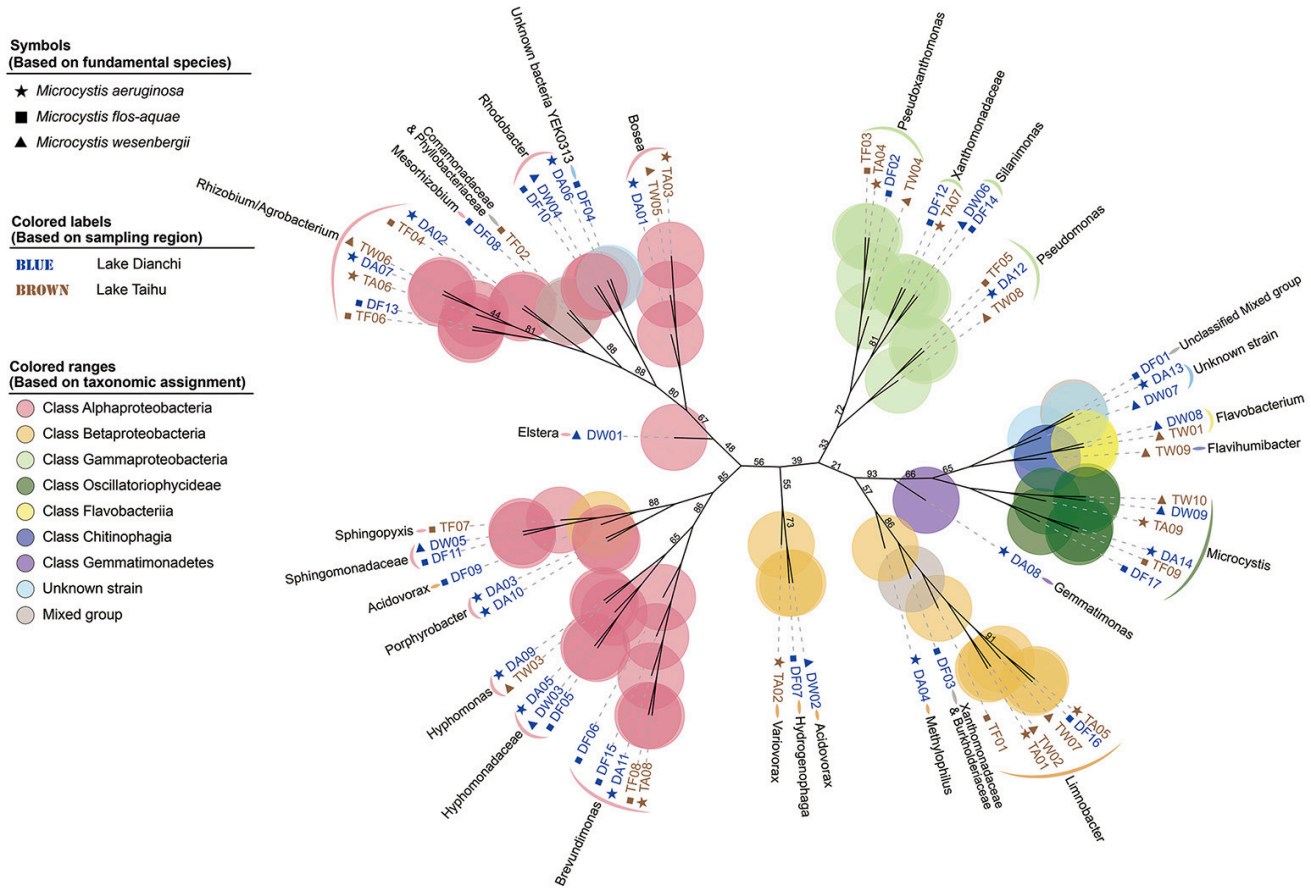
Genome-based phylogenetic inference of 68 strains. A phylogenetic tree, inferred with composition vectors and the neighbor-joining model, based on whole predicted proteome sequences of each genome bin revealing taxa at the genus level. *Microcystis* colonies were sampled from Lake Dianchi (blue) or Lake Taihu (brown). *Microcystis aeruginosa*-, *M. flos-aquae*-, and *M. wesenbergii*-dominated communities are marked with stars, squares, and triangles, respectively. The values on the nodes represent percentages of bootstrap confidence levels <95% from 1,000 replicates.

Based on protein-encoding gene similarity, a genome-wide tree of microbes (Figure 2) showed that bacteria identified as the same taxon clustered closely together, regardless of their origin. Overall, in the six *Microcystis*-epibiont communities, the diversity of Alphaproteobacteria was high, and bacteria of at least nine alphaproteobacterial families were observed. For Betaproteobacteria, *Limnobacter* was found in four of the communities. Only two families of Gammaproteobacteria were detected in the communities, and they clustered together in the tree. In terms of subclades with the same taxonomic identification, 14 taxa contained more than one strain that shared highly similar genomes and were regarded as common groups in *Microcystis*-epibiont communities; these taxa included genera *Brevundimonas, Hyphomonas, Porphyrobacter, Rhodobacter, Bosea, Limnobacter, Pseudomonas, Pseudoxanthomonas, Silanimonas, Flavobacterium*, and families Hyphomonadaceae, Sphingomonadaceae, Xanthomonadaceae, and the Rhizobium/Agrobacterium group. The presence of common groups is consistent with the pairwise genome comparisons results based on the average nucleotide identity value (Supplementary Figure S4). It should be noted that among these common groups, *Rhodobacter* spp. and *Hyphomonadaceae* spp. were only found in the three communities isolated from Lake Dianchi, and *Flavobacterium* spp. were only detected in the two *M. wesenbergii*-dominated communities (DW, TW), whereas *Brevundimonas* was only found in the other four communities (DA, DF, TA, TF).

### 3.5. Pathway reconstruction demonstrates complex metabolic capabilities of the *Microcystis* microbiome

**Figure 3:**
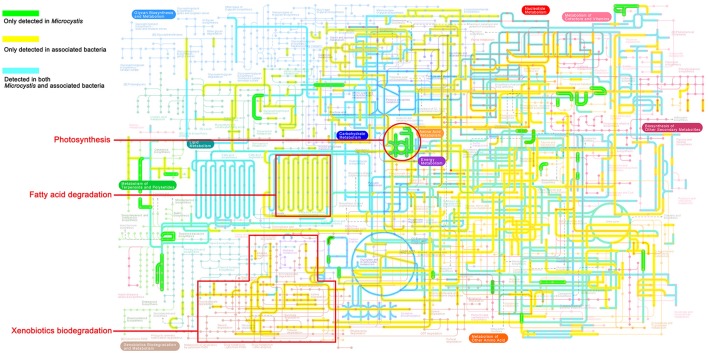
KEGG map of metabolic pathways present in the *Microcystis*-epibiont communities (data for sample TW is shown as an example). The highlighted colored lines show metabolic pathways detected in the community. Green lines represent pathways exclusively detected in *Microcystis*, yellow lines represent pathways exclusively detected in epibionts, and blue lines represent pathways detected in both *Microcystis* and their epibionts.

As shown in the pathway maps reconstructed by GhostKOALA (Figure 3), *Microcystis* and epibionts both contributed to multiple molecular interactions and reaction networks in KEGG pathways, including energy metabolism, carbohydrate and lipid metabolism, nucleotide and amino acid metabolism, and secondary metabolism. Moreover, differences between pathways in *Microcystis* and epibionts highlighted the important functions of each for prosperity of the whole community. *Microcystis* is the only species capable of photosynthesis as a primary producer, and provides carbon and energy to heterotrophic bacteria in the community. However, enzymes responsible for aromatic compound degradation and fatty acid catabolism were only identified in epibiont genomes.

### 3.6. Potential interactions of biogeochemical nitrogen and sulfur cycling in the *Microcystis* microbiome

**Figure 4:**
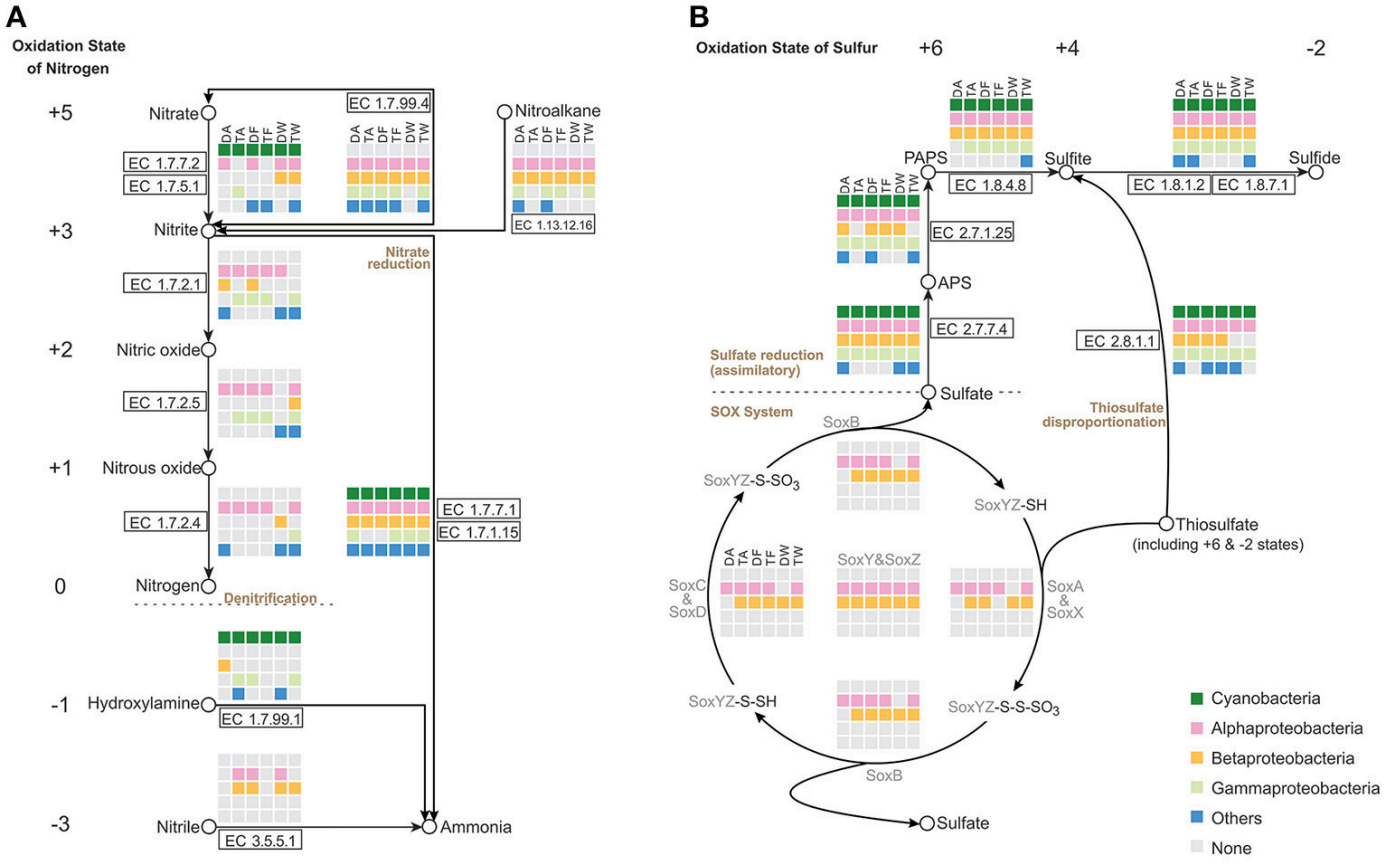
Nitrogen and sulfur metabolic pathways identified in the six *Microcystis*-epibiont communities. **(A)** Nitrogen metabolism; **(B)** Sulfur metabolism. Diagrams are based on KEGG pathway maps. Available enzyme classification numbers for each step are included in boxes. Each column represents a different community. DA, TA, DF, TF, DW, and TW are the abbreviations for the six *Microcystis*-epibiont communities. Box colors indicate bacteria from different classes: dark green, Cyanobacteria (*Microcystis*); pink, Alphaproteobacteria; gold, Betaproteobacteria; light green, Gammaproteobacteria; and blue, other phyla such as Bacteroidetes or Gemmatimonadetes. Gray indicates absence of the corresponding enzyme from the community.

Focusing on the metabolism of essential elements, we analyzed the relevant pathways that showed conversion of nitrogen or sulfur through various oxidation states and participation of major groups in the *Microcystis*-epibiont communities (Figure 4).

Nitrogen is a fundamental constituent of biomolecules, and its oxidation states range from +5 to −3 in different compounds. Denitrification converts nitrate and nitrite into nitrogenous gases, and thus removes fixed nitrogen from the biosphere. In each of our samples, all functional genes required for denitrification were found (Figure 4A), including the genes that encode enzymes NarGHI (EC1.7.5.1), NapAB (EC1.7.99.4), NirK (EC1.7.2.1), NirS (EC1.7.2.1), NorBC (EC1.7.2.5), and NosZ (EC1.7.2.4). Alphaproteobacteria and Betaproteobacteria tended to be responsible for denitrification in the communities. With the enzymes NirA (EC1.7.7.1) or NirBD (EC1.7.1.15), both *Microcystis* and epibionts can achieve nitrite reduction (Figure 4A). We did not find enzymes for nitrogen fixation in the metagenomes of the *Microcystis*-epibiont communities.

Metabolism of sulfur compounds, with oxidation states that range from +6 to −2, plays an important role in the global sulfur cycle. Identification of genes related to sulfur metabolism pathways revealed that *Microcystis* and epibionts were both involved in assimilatory sulfate reduction (ASR) (Figure 4B). Thiosulfate acts as a relatively stable sulfur compound enriched in the environmental sulfur cycle; this compound has two sulfur atoms with respective oxidation states of +6 and −2. The Sox enzyme system can catalyze the direct oxidation of thiosulfate to sulfate with no intermediates. Some Alphaproteobacteria and Betaproteobacteria in the six samples possessed the sox gene cluster, which includes soxXYZABCD (Figure 4B); this indicates that heterotrophic bacteria in the *Microcystis*-epibiont communities have the capacity for thiosulfate oxidation. Both *Microcystis* and epibionts may perform thiosulfate disproportionation via rhodanese (EC 2.8.1.1) (Figure 4B), which is an enzyme that catalyzes the transfer of a sulfur atom from suitable donors to nucleophilic sulfur acceptors.

### 3.7. Epibionts oxidize fatty acids to provide carbon and energy for cell growth

**Figure 5:**
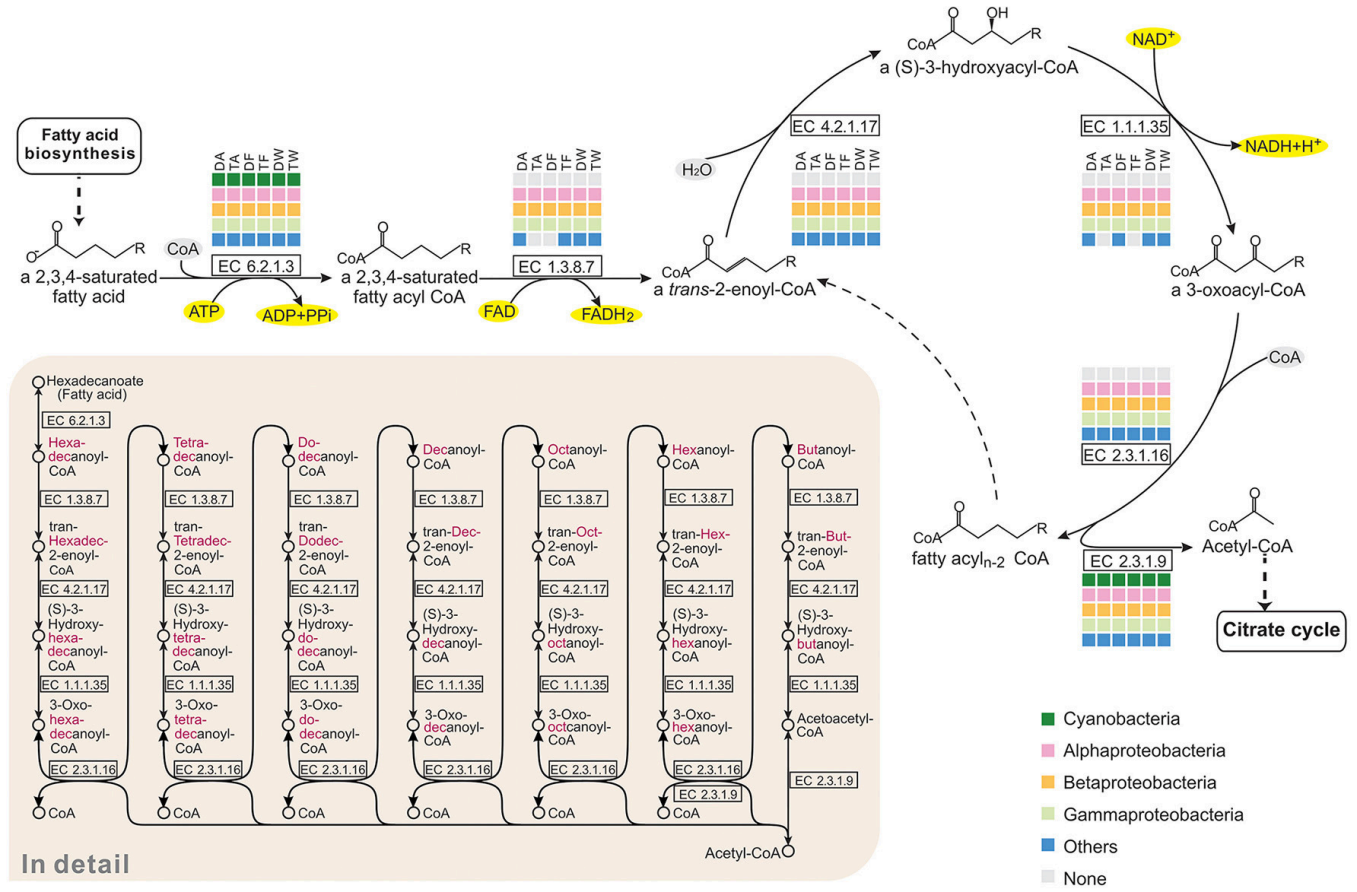
Comparison of the KEGG fatty acid degradation pathways of the six *Microcystis*-epibiont communities. The figure represents the core cycle of β-oxidation, which is a classical route that removes two carbon atoms with each turn of the cycle as fatty acid catabolism proceeds. Available enzyme classification numbers for each step are included in boxes. Each column represents a different community. DA, TA, DF, TF, DW, and TW are the abbreviations for the six *Microcystis*-epibiont communities. Box colors indicate bacteria from different classes: dark green, Cyanobacteria (*Microcystis*); pink, Alphaproteobacteria; gold, Betaproteobacteria; light green, Gammaproteobacteria; and blue, other phyla such as Bacteroidetes or Gemmatimonadetes. Gray indicates absence of the corresponding enzyme from the community.

In the pathway maps (Figure 3), there was a clear difference between *Microcystis* and the epibionts in fatty acid catabolism. Figure 5 shows the participation of community members in β-oxidation, which removes two carbon atoms with each turn of the cycle in fatty acid degradation. In the six communities, Alphaproteobacteria, Betaproteobacteria, Gammaproteobacteria, and some other bacteria possess all the genes required for β-oxidation, but none of these genes were found in *Microcystis*. All the associated bacterial groups have the potential to produce acetyl-CoA by breaking down even-numbered fatty acids, and the final product, acetyl-CoA, can be fed into the citrate cycle (TCA cycle) for complete catabolism or routed into the glyoxylate cycle for biosynthesis. This is an advantageous pathway for epibionts to provide carbon and energy for cell growth.

### 3.8. Epibionts cooperatively degrade aromatic compounds

**Figure 6:**
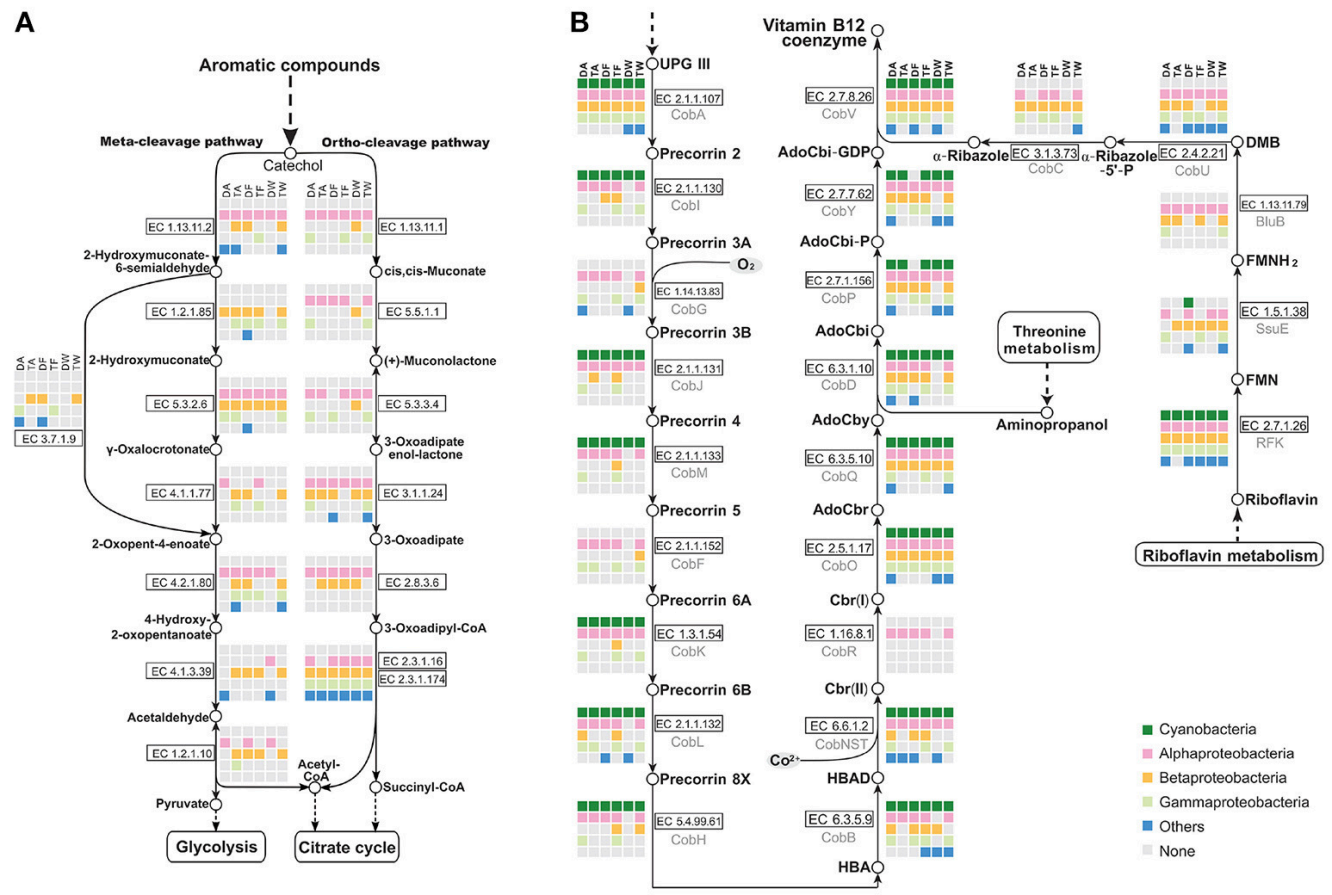
Analysis of metabolic pathways among the six *Microcystis*-epibiont communities. **(A)** Aromatic compound catabolism pathways; **(B)** Aerobic biosynthesis pathways of vitamin *B*_12_. Diagrams are based on KEGG pathway maps. Available enzyme classification numbers for each step are included in boxes. Each column represents a different community. DA, TA, DF, TF, DW, and TW are abbreviations for the six *Microcystis*-epibiont communities. Box colors indicate bacteria from different classes: dark green, Cyanobacteria (*Microcystis*); pink, Alphaproteobacteria; gold, Betaproteobacteria; light green, Gammaproteobacteria; and blue, other phyla such as Bacteroidetes or Gemmatimonadetes. Gray indicates absence of the corresponding enzyme from the community.

Metabolic pathway reconstruction and network analysis produced evidence of a functional complementation among *Microcystis*-epibiont community members. The cleavage pathway of catechol is one of the major pathways for the degradation of aromatic compounds. Based on analysis of functional genes, epibionts in each community were determined to carry out different steps in the pathway to assist in catechol degradation to yield acetyl-CoA, pyruvate, and succinyl-CoA, in contrast *Microcystis* is not directly involved in this process (Figure 6A).

### 3.9. Epibionts contribute to vitamins synthesis

There is additional evidence in the pathway maps showing that relationships among community members are mutually beneficial. For example, several individual epibionts in the community cooperate in the aerobic biosynthesis pathway of adenosylcobalamin (also known as vitamin *B*_12_ or coenzyme *B*_12_) (Figure 6B). *Microcystis* has the methionine biosynthesis pathway, which includes a type-II MetH enzyme that requires vitamin *B*_12_ as a cofactor. This was reported in earlier research (Helliwell et al., 2011; Xie et al., 2016) and was supported by a BLAST search of all sequenced *Microcystis* genomes in the NCBI database. Without vitamin *B*_12_ as an additive in the medium, *Microcystis*-epibiont community subcultures still grew, probably owing to vitamin *B*_12_ or methionine produced by other community members. The pathway analysis showed that six enzymes, CobG (EC 1.14.13.83), CobR (EC 1.16.8.1), BluB (EC 1.13.11.79), CobT (EC 2.4.2.21), CobC (EC 3.1.3.73), and CobF (EC 2.1.1.152), were absent from the six *Microcystis* genomes. This result supports the idea that the *Microcystis* strains in our samples were unable to synthesize vitamin *B*_12_ *de novo*. However, the microbiome consortia in each community could complete vitamin *B*_12_ biosynthesis. It is likely that at least one epibiont contributed to vitamin *B*_12_ synthesis in each community, which would benefit *Microcystis*. Similar complementation was also discovered in the pathways for synthesis of other cofactors, such as riboflavin (vitamin *B*_2_) and biotin (vitamin *B*_7_).

### 3.10. Functional potential of the whole community and its relation to the original environment

**Figure 7:**
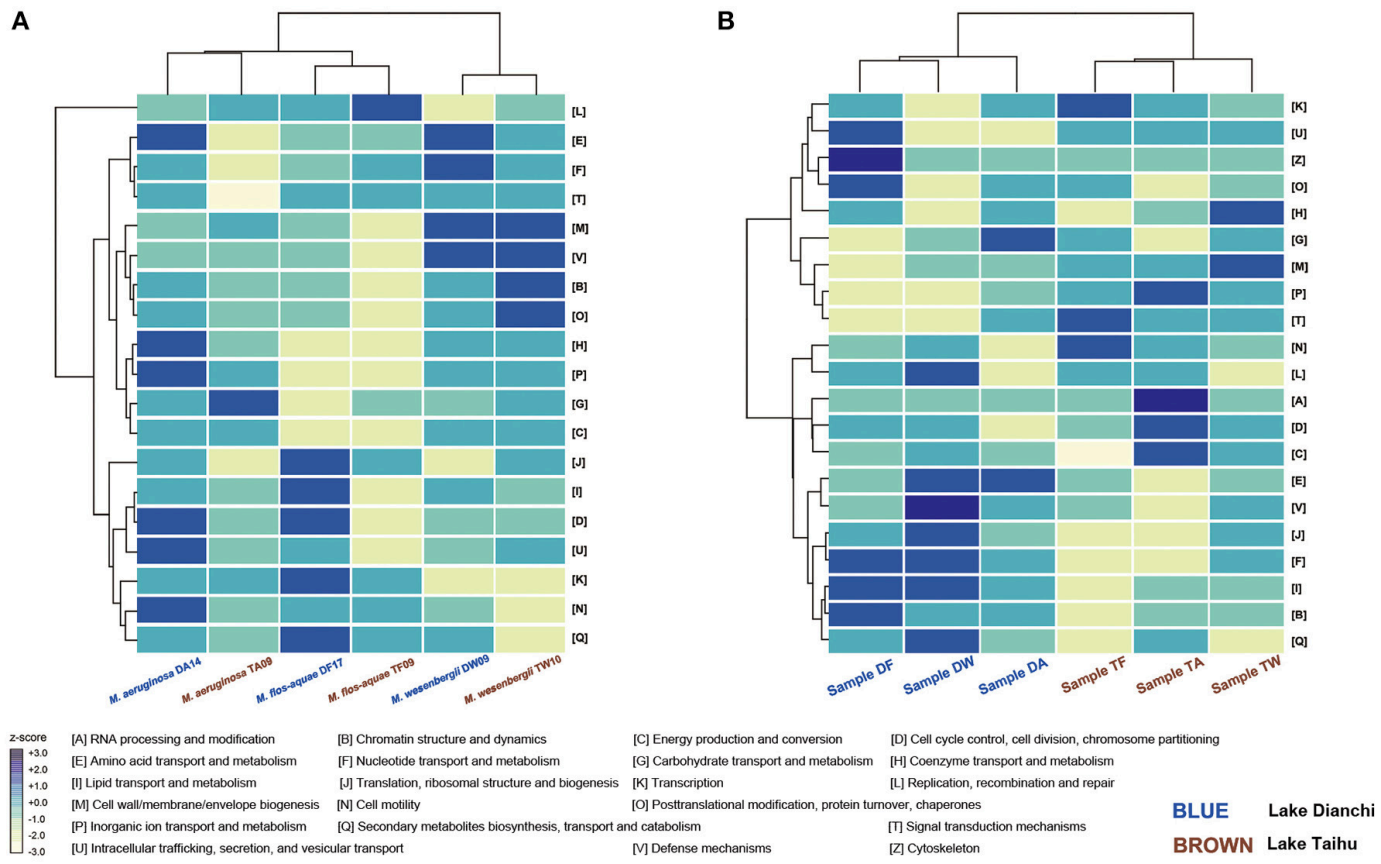
Functional profiling of *Microcystis* and *Microcystis*-epibiont communities. **(A)** Heatmap that shows two-way clustering of COG profiles from the *Microcystis* genomes. **(B)** Heatmap that shows two-way clustering of COG profiles from genomes of *Microcystis*-epibiont communities that originated from Lake Dianchi and Lake Taihu. COG categories for each row are displayed on the right side of each heatmap.

The normalized proportion of proteins in each COG category was plotted vs. the COG function to elucidate the different functional roles of the community members (Figure 7). Considering only the six *Microcystis* spp. genomes, functional heatmaps highlighted that strains of the same morphospecies clustered together, whereas different morphospecies showed differences in their functional genome profiles (Figure 7A). In particular, defense mechanisms (V) and cell wall/membrane/envelope biogenesis (M) were the most abundant functional categories in *M. wesenbergii*. Genes involved in defense mechanisms (V) enhance microbe adaptability to diverse ecological niches. The prevalence of genes involved in cell wall/membrane/envelope biogenesis indicates that *M. wesenbergii* might show some biological differences in cell wall properties from *M. aeruginosa* and *M. flos-aquae*.

Taking both *Microcystis* and epibionts into account, the communities from Lake Taihu gathered in one clade, whereas the samples from Lake Dianchi formed another clade (Figure 7B). Several significant differences of orthologous protein groups between the microbiomes of these two different geographic origins were observed by examining individual genetic and functional distributions in detail (Supplementary Figures S5, S6).

## 4. Discussion

Due to the industrialization and global warming, harmful cyanobacterial blooms are resurging worldwide, with serious effects on human health, the economy, and the environment (Falconer, 2001; Hilborn et al., 2014; Otten and Paerl, 2015). To effectively manage and control cyanobacterial blooms, especially *Microcystis*-based blooms, it is imperative for us to understand the fundamental mechanisms that make *Microcystis* the dominant species in eutrophic water. *Microcystis* colonies harbor epibionts that establish mutualistic interactions in the community. Bacterial social interactions shape not only the phenotypes of each community member, but also the composition and functional profile of the whole community (Madsen et al., 2018). The functional complementation and synergies among *Microcystis* and epibionts may impact the metabolic capabilities of the whole community. In this work, we performed a binning-based metagenomics approach to investigate community structure, species diversity, functional components, metabolic pathways, and networks of six *Microcystis*-epibiont communities isolated from two lakes.

### 4.1. Construction of a *Microcystis* microbiome reference genome catalog

The integrated metagenomic pipeline, which consisted of efficient assembly, binning, annotation, and quality assurance methods, facilitated high-quality genome reconstruction and identification. Sixty-eight microbial genomes were reconstructed from metagenomic data of the six samples, including six complete *Microcystis* genomes and 28 high-quality bacterial genomes of epibionts. The epibionts belonged to 14 distinct taxa, of which two are new bacterial species that were not previously reported. The reference genome catalog provides detailed information on species composition and bacterial social interactions of the *Microcystis*-epibiont communities. This information will greatly facilitate future genome-centric analyses of natural microbial communities that originated from Lake Taihu and Lake Dianchi.

### 4.2. *Microcystis*-epibiont community is dynamic in species composition

To focus on the interaction between *Microcystis* cells and the bacteria that are tightly embedded in their peripheral muciferous layer, we processed samples with multiple washing and cultivation steps. A previous study by Zhu et al. (2016) showed that there were no strong differences in richness or diversity between the natural and cultivated cyanobacteria associated bacteria communities, even in some strains isolated 11 years earlier. Taking advantage of the metagenomic dataset, we investigated the community structure and species diversity within the *Microcystis* microbiome. The metagenomic data revealed that the community consisted of *Microcystis* and dominant epibionts (Alphaproteobacteria, Betaproteobacteria, and Gammaproteobacteria) that were commonly present in all of our samples, and other bacteria species that were unique to some morphospecies and/or samples from a specific lake (Figure 2 and Supplementary Table S3). Sphingomonadales, Xanthomonadaceae, and Burkholderiales were abundant bacterial groups in our *Microcystis*-epibiont communities, which were also commonly found in freshwater ecosystems (Berg et al., 2009; Limei et al., 2009; Dziallas and Grossart, 2011; Shen et al., 2011; Shi et al., 2012). Within the Alphaproteobacteria, Sphingomonadales species are capable of degrading polysaccharides, microcystins, hexachlorocyclohexane, and various other organic compounds (Valeria et al., 2006; Ho et al., 2007; Bala et al., 2010), and Rhodobacterales are involved in the degradation of aliphatic and aromatic hydrocarbons (Harwati et al., 2007; Brinkhoff et al., 2008). Within the Betaproteobacteria, *Limnobacter* spp. presumably degrades a vast array of aromatic compounds (Pérez-Pantoja et al., 2012; Vedler et al., 2013). Within the Gammaproteobacteria, *Pseudomonas* spp. were observed undertaking phosphorus exchange with *M. aeruginosa* (Jiang et al., 2007), and Xanthomonadaceae strains were detected to be involved in petroleum hydrocarbon degradation (Chang and Zylstra, 2010). Members of the Bacteriodetes contribute to high-molecular-weight organic matter degradation (Cottrell and Kirchman, 2000).

The characteristics of epibionts imply that biosynthesis and release of toxins and complex organic compounds may be balanced by the biodegradation of these harmful pollutants in the microenvironment. In the samples isolated from both lakes, *Flavobacterium* was only detected in the *M. wesenbergii* colonies, whereas *Brevundimonas* was only found in the other *Microcystis* morphotypes. Both *Flavobacterium* spp. and *Brevundimonas* spp. were found to be capable of degrading complex organic compounds (Konno et al., 2006; Valeria et al., 2006; Tumaikina et al., 2008; Berg et al., 2009). These results might reflect the conditions of Lake Taihu and Lake Dianchi, which are heavily loaded with organic matter and environmental pollutants. Furthermore, two nearly complete genomes with high abundance in the communities isolated from Lake Dianchi did not match any previous genome sequence records, implicating that these unknown bacteria might depend on *Microcystis* strains for their existence and are difficult to identify or culture. No species was shared by all six samples, which demonstrates that specific, closely associated heterotrophic bacterial species is not indispensable for *Microcystis* growth. The species composition of epibionts appear to be dynamic rather than fixed in the communities, varying according to the morphospecies or the origin.

### 4.3. *Microcystis* and epibionts both benefit from functional interactions

To study the bacterial social interactions within the six *Microcystis*-epibiont communities, organism-specific metabolic pathways were reconstructed in the KEGG pathway maps (Figure 3). Pathways involved in photosynthesis were only detected in *Microcystis*, which is consistent with a previous report that *Microcystis* is the only photoautotrophic bacterium in the community (Cole, 1982). Carbon, nitrogen, sulfur, and phosphorus are fundamental constituents of biomolecules. The microbial communities here had diverse sets of enzymes for cycling through various oxidation states of nitrogen and sulfur compounds with different physicochemical properties (Figure 4), which indicates that the associated bacteria are essential for maintaining the microenvironmental redox balance and influencing the nitrogen and sulfur cycles.

Fatty acid catabolism and the energy efficiency of fatty acid degradation, which involve the citrate cycle or glyoxylate cycle, were processed in the community microenvironment rather than by *Microcystis* (Figure 5); this indicates that the associated bacteria play important roles in fatty acid degradation and might obtain both carbon and energy from this process with acyl-CoA synthesis. Our metabolic analysis showed that aromatic compounds, such as benzoate, are likely degraded through cooperative actions of the epibionts (Figure 6A). Furthermore, vitamin *B*_12_ biosynthesis pathway analysis demonstrated that one or more of the associated bacteria, especially alphaproteobacterial species, contribute to vitamin *B*_12_ synthesis in the microenvironment and supply vitamin *B*_12_ for *Microcystis* growth (Figure 6B). A previous study of *M. wesenbergii* T100 community also suggested that individual bacteria in the community contributed a complete pathway for the biosynthesis of Vitamin *B*_12_ and degradation of benzoate (Xie et al., 2016). A similar mutualism based on vitamin *B*_12_ and nutrient exchange was described in an artificial coculture of *Synechococcus* sp. PCC 7002 and two heterotrophic bacteria (Ren et al., 2017). Bacterial social interactions among community members, such as extracellular electron transfer, energy flux, and material circulation, are ubiquitous in *Microcystis*-epibiont communities.

### 4.4. Functional profiles of the *Microcystis*-epibiont communities are associated with the environment of origin

Using the integrated binning method, we were able to explore the functional attributes of each *Microcystis* strain. Cluster analysis of functional profiles of the genomes of the six *Microcystis* strains showed that profiles of the same morphospecies isolated from different lakes were more similar than those of the other morphospecies isolated from the same lake (Figure 7A), indicating that morphospecies corresponded to similar genotypes. Specifically, *M. wesenbergii* strains have a higher abundance of genes linked to defense mechanisms and cell wall/membrane/envelope biogenesis compared with other species studied. These results might help explain previous observations (Werner et al., 2015) that *M. wesenbergii* is easily distinguished from other species of the genus by the characteristic outline of its colonies.

A previous study demonstrated that the predicted functional genetic complement of a microbial community was similar to that of other communities originated from environments with similar metabolic requirements (Tringe et al., 2005). For example, there was a clear relationship between bacterial community structure and the eutrophication state of the environment (Liu et al., 2009; Wen et al., 2012). Nutrient contents in Lake Dianchi were much higher than those in Lake Taihu, whereas the range of water temperature and dissolved oxygen in Lake Taihu were much wider than those in Lake Dianchi (Figure 1A) (Huang et al., 2010; Niu et al., 2011; Cao et al., 2012). Cluster analysis using functional profiles of the six *Microcystis*-epibiont communities resulted in two groups, with samples isolated from the same lake clustered together (Figure 7B). From the perspective of the distribution of genetic and functional profiles, several significant differences in orthologous protein groups were observed between samples isolated from the two different lakes (Supplementary Figures S5, S6). For example, some COGs for defense mechanisms (V) were present in the three samples isolated from Lake Taihu and absent from the Lake Dianchi samples. Some COGs for secondary metabolites biosynthesis, transport and catabolism (Q), and energy production and conversion (C) were only present in the three samples isolated from Lake Dianchi.

Our results indicate that the functional profiles of *Microcystis*-epibiont communities are influenced by their environment of origin more than by the dominant *Microcystis* species in the community. Even if the strains had been maintained in culture for various periods of time, characteristic functional profiles of each community could still be distinguished. These results can help us understand the relevance of *Microcystis*-epibiont interactions to the success of *Microcystis* strains in their original environments.

## 5. Conclusions

A reference genome catalog of the *Microcystis* microbiome will greatly facilitate genome-centric analysis of natural microbial communities in bloom-occurring lakes. Binning-based metagenomic analysis revealed that functional complementation and synergistic interactions were ubiquitous in all six *Microcystis*-epibiont communities. Although the species composition of the six microbiomes differed from each other, their functional profiles were related to the environment where they originated. Bacterial social interactions within *Microcystis*-epibiont communities are reflected not only in species composition but also in the pattern of metabolic functions. These traits may play an important role in enabling *Microcystis* colonies to maintain a competitive advantage in hypereutrophic lakes.

## Author contributions

QL and FL carried out the analysis of data and prepared the first draft of the manuscript; TL and JZ helped design the project and revised the manuscript; CY assisted in sample collection; JW and YL performed the sequencing; MS assisted in data analysis; MP revised and polished the manuscript. All authors participated in the discussion of manuscript and have agreed to the final content.

### Conflict of interest statement

The authors declare that the research was conducted in the absence of any commercial or financial relationships that could be construed as a potential conflict of interest.
