# Supplementary material for: A Large-Scale Comparative Metagenomic Study Reveals the Functional Interactions in Six Bloom-Forming Microcystis-Epibiont Communities
Source: Front Microbiol. 2018 Apr 20;9:746. doi: 10.3389/fmicb.2018.00746 (PMC5919953; doi:10.3389/fmicb.2018.00746)
Supplement: Supplementary file 1 [file Presentation1.PDF]

Supplementary Material

A large-scale comparative metagenomic study reveals the functional interactions in six bloom-forming *Microcystis*-epibiont communities

Qi Li [†], Feibi Lin [†], Chen Yang, Juanping Wang, Yan Lin, Mengyuan Shen, Min S. Park, Tao Li * and Jindong Zhao

*** Correspondence:** Tao Li: litao@ihb.ac.cn

[†] These authors have contributed equally to this work.

File content

This file contains **Supplementary Figures S1-S6**.

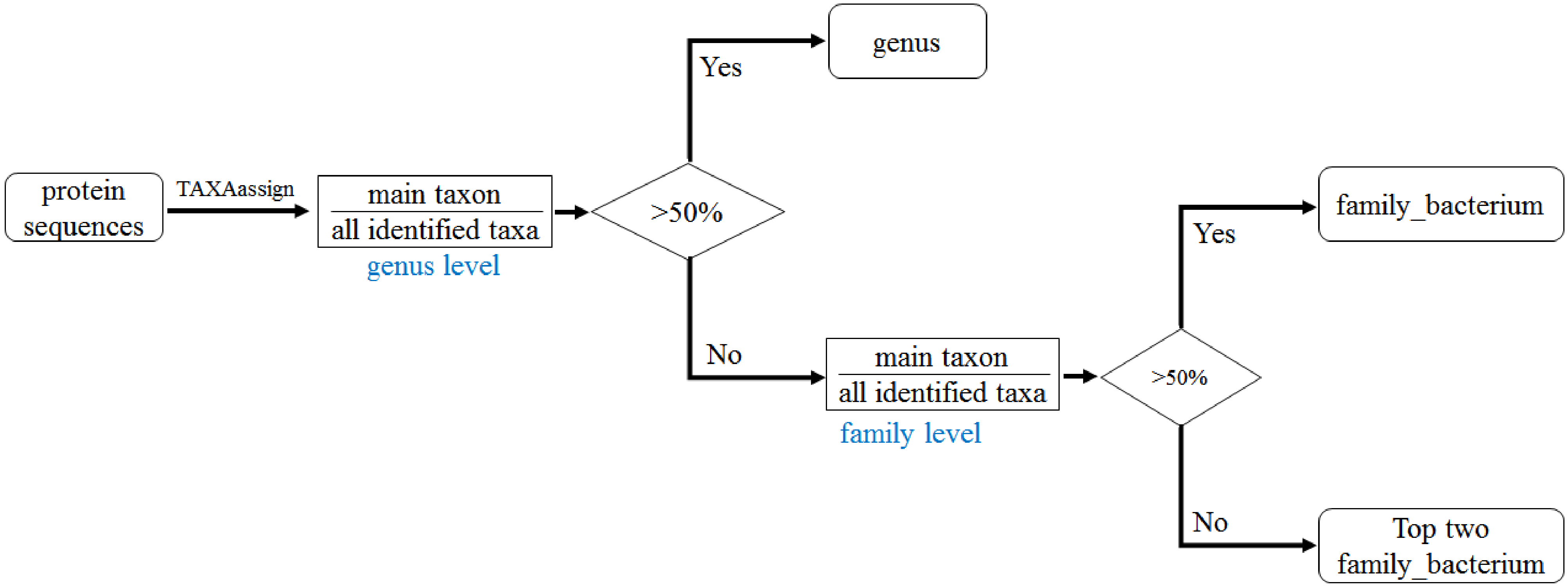

Supplementary Figure S1. Flow chart of the taxonomic classification protocol.

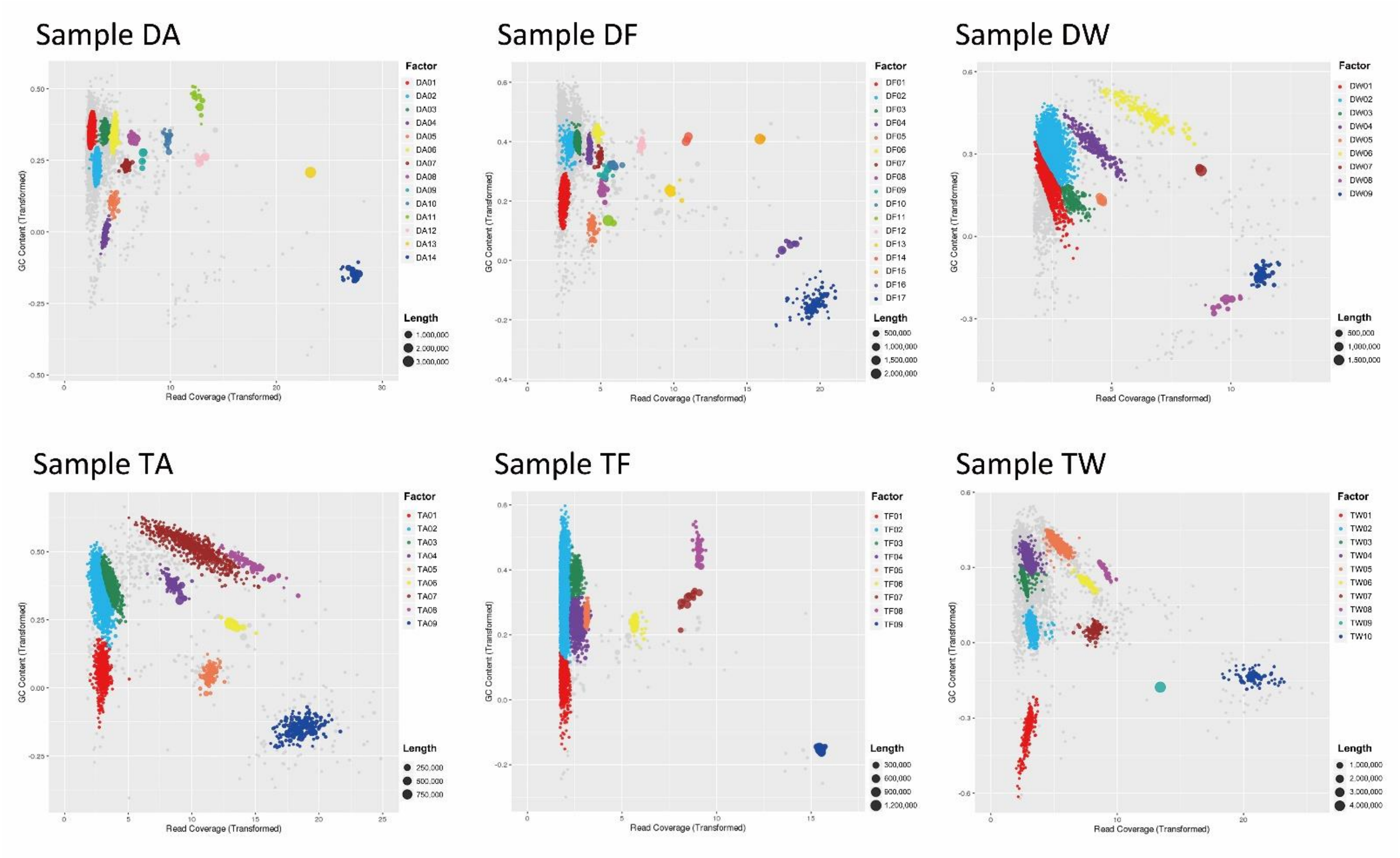

Supplementary Figure S2. Two-dimensional scatterplot of the binning results for the six *Microcystis*-epibiont communities. The points represent scaffolds assembled from the metagenomic sequence data sets and the point size indicates the length of the scaffold. The different colored clusters on the graph are potential different genome bins.

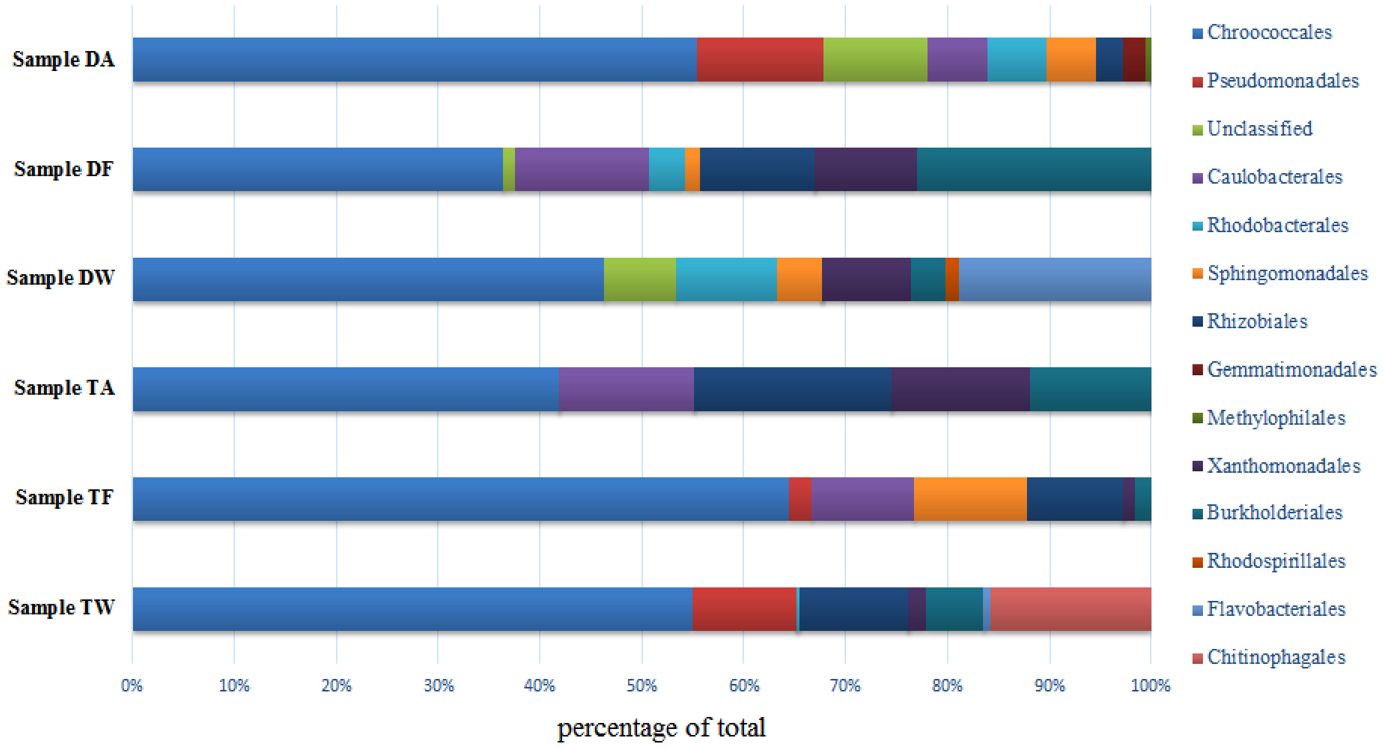

Supplementary Figure S3. Relative abundance (percentage) of major bacterial orders in each *Microcystis*-epibiont community. *Microcystis* (order *Chroococcales*) was the most abundant group in each sample.

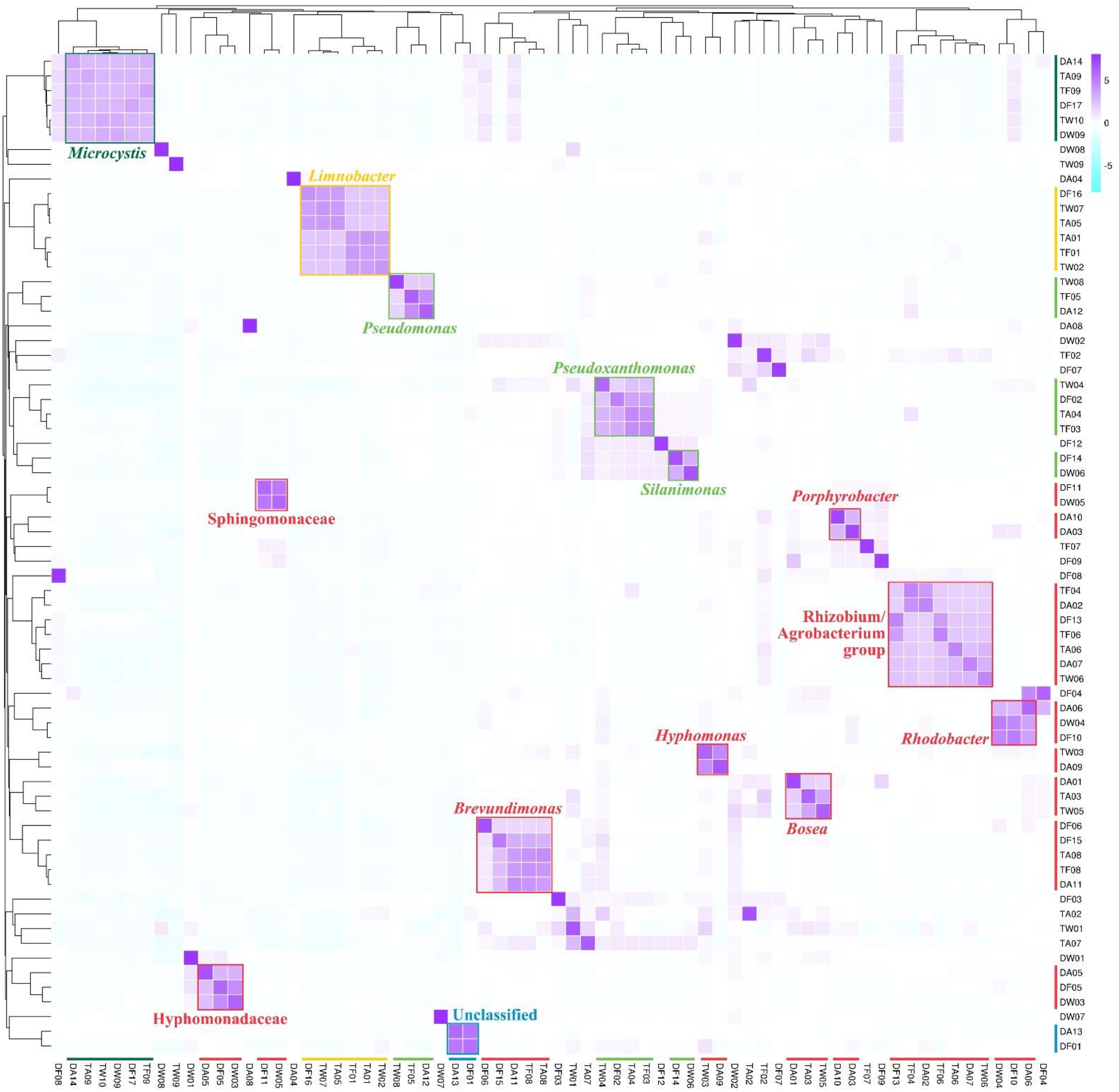

Supplementary Figure S4. Average Nucleotide Identity (ANI) value heatmap with a normalized hierarchical clustering of the 68 genome bins. The cell colors represent the normalized ANI values.

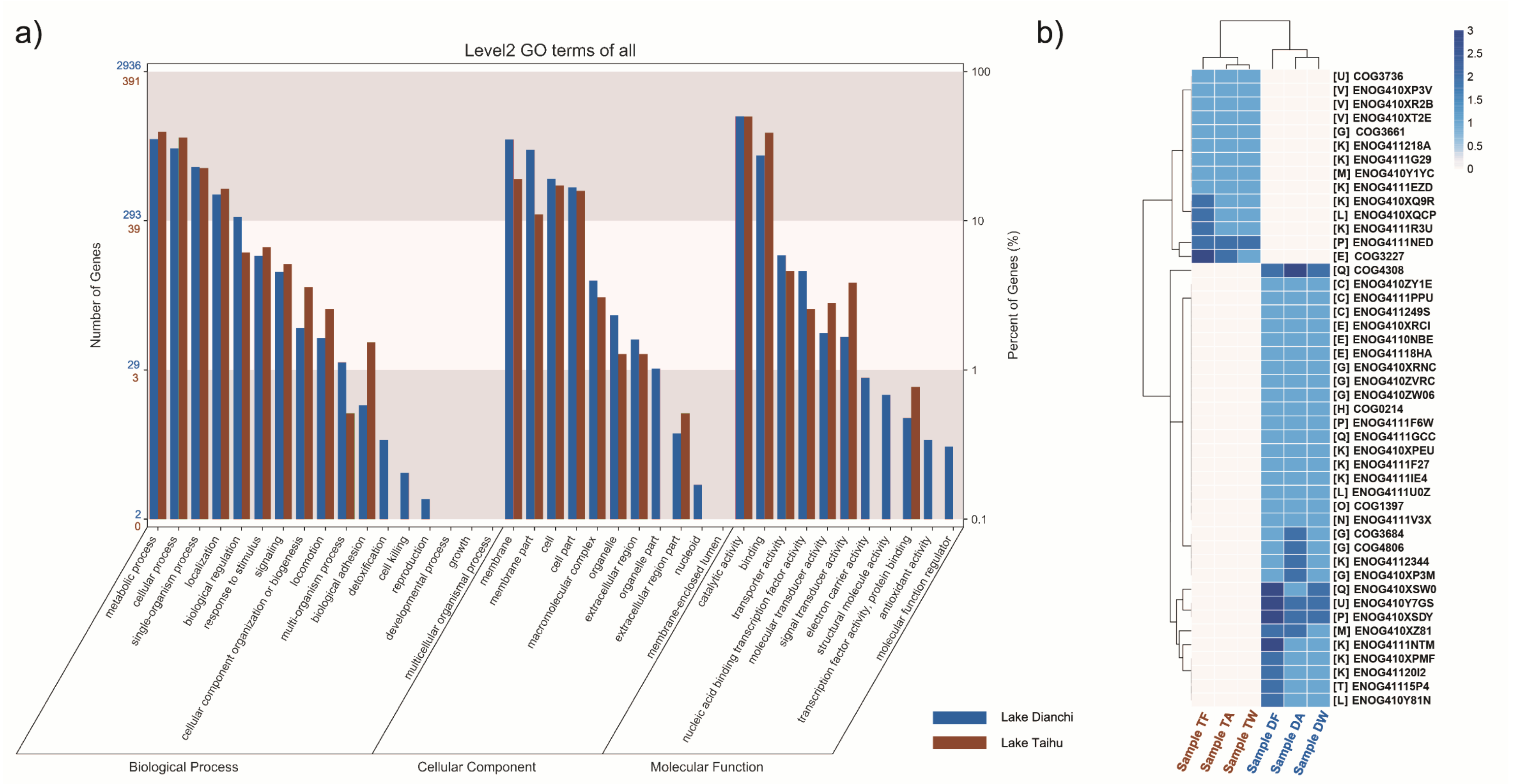

Supplementary Figure S5. Genetic and functional analysis of different orthologous protein groups in six *Microcystis*-epibiont communities. (a) Gene Ontology (GO) enrichment analysis of specific orthologous protein groups of communities isolated from different lakes. The blue bars represent orthologous protein groups exclusively detected in the communities isolated from Lake Dianchi, the brown bars represent orthologous protein groups exclusively detected in the communities isolated from Lake Taihu. Orthologous proteins that detected in the communities isolated from both lakes have not been included. (b) COGs analysis of specific orthologous protein groups of communities isolated from different lakes.

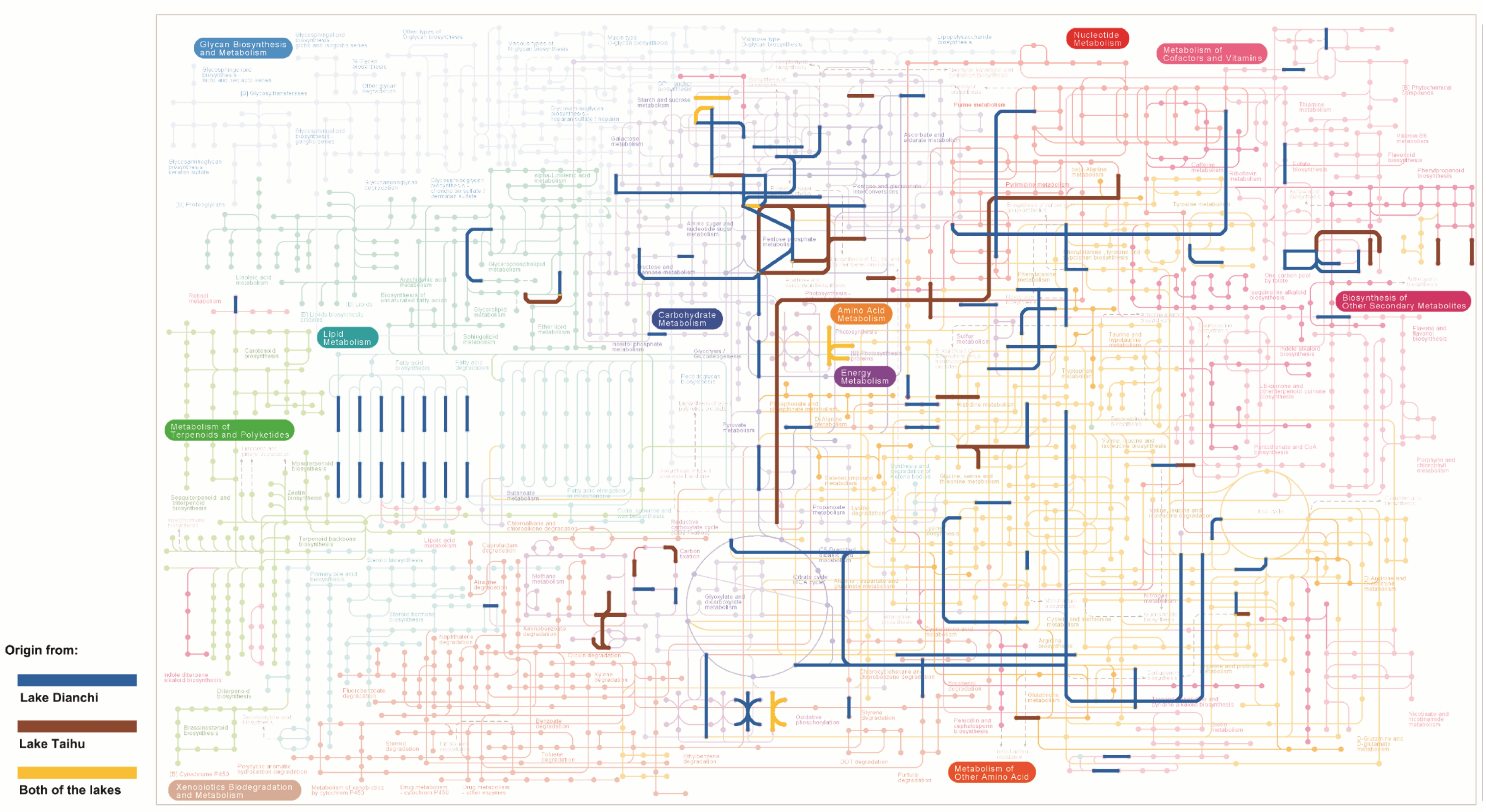

Supplementary Figure S6. Metabolic pathways analysis of different orthologous protein groups in the *Microcystis*-epibiont communities. The blue lines represent pathways exclusively detected in specific orthologous protein groups of communities isolated from Lake Dianchi, the brown lines represent pathways exclusively detected in specific orthologous protein groups of communities isolated from Lake Taihu, and yellow lines represent pathways detected in specific orthologous protein groups of communities isolated from both lakes.
